# A Wind Energy Powered Wireless Temperature Sensor Node

**DOI:** 10.3390/s150305020

**Published:** 2015-02-27

**Authors:** Chuang Zhang, Xue-Feng He, Si-Yu Li, Yao-Qing Cheng, Yang Rao

**Affiliations:** 1 Key Laboratory of Optoelectronic Technology and Systems of the Education Ministry of China, Chongqing University, Chongqing 400044, China; E-Mails: 20120802066@cqu.edu.cn (C.Z.); dalisiyu@126.com (S.-Y.L.); engineerao@163.com (Y.R.); 2 Microsystem Research Center, Chongqing University, Chongqing 400044, China; E-Mail: chengyaoqing07@163.com

**Keywords:** wireless sensor node, temperature, energy harvesting, wind-induced vibration, piezoelectricity

## Abstract

A wireless temperature sensor node composed of a piezoelectric wind energy harvester, a temperature sensor, a microcontroller, a power management circuit and a wireless transmitting module was developed. The wind-induced vibration energy harvester with a cuboid chamber of 62 mm × 19.6 mm × 10 mm converts ambient wind energy into electrical energy to power the sensor node. A TMP102 temperature sensor and the MSP430 microcontroller are used to measure the temperature. The power management module consists of LTC3588-1 and LT3009 units. The measured temperature is transmitted by the nRF24l01 transceiver. Experimental results show that the critical wind speed of the harvester was about 5.4 m/s and the output power of the harvester was about 1.59 mW for the electrical load of 20 kΩ at wind speed of 11.2 m/s, which was sufficient to power the wireless sensor node to measure and transmit the temperature every 13 s. When the wind speed increased from 6 m/s to 11.5 m/s, the self-powered wireless sensor node worked normally.

## 1. Introduction

Small-scale or micro-scale energy harvesters, which convert ambient energy into electrical energy, are ideal electrical sources of wireless sensor nodes due to advantages as small volumes, long lives, maintenance-free and so on. In recent years, a variety of energy harvesters have been developed to scavenge ambient energy sources, such as solar, vibration or wind energy, to power wireless sensor nodes. Vibration energy harvesters have received much attention due to the ubiquitous presence of the vibration in the environment [1,2,3,4,5]. In the past few years, more and more efforts have been devoted to wind energy harvesters [6,7,8,9,10,11,12,13,14,15,16,17]. There are mainly two types of small-scale or micro-scale wind energy harvesters. The first one is turbine energy harvesters [6,7,8], which were typically composed of blades, magnets, coils and other parts. It is difficult to miniaturize these devices by MEMS technology. The other is flow-induced vibration or wind-induced vibration energy harvesters [1,9,10,11,12,13,14,15,16,17,18,19,20,21,22], whose structures are relatively simple and can be miniaturized by micromachining process. The airflow causes beams or diaphragms to vibrate, and the vibration energy is then converted into electrical energy based on mechanical-to-electrical conversion mechanisms such as piezoelectric effect, electromagnetic induction or electrostatic induction.

A large number of wind-induced vibration piezoelectric energy harvesters have been reported in literatures. A wind energy harvester composed of a piezoelectric cantilever with the dimension of 254 mm × 25.4 mm and a tip flap with the width of 136 mm was reported [15]. The maximum output power was about 2.2 mW. Another piezoelectric cantilever with the dimensions of 30 mm × 16 mm × 0.2 mm placed in the wake of a circular cylinder produced the maximum output power of about 4 µW [16]. To power a wireless sensor node for heating, ventilation and air conditioning monitoring systems, a wind-induced vibration piezoelectric energy harvester with the dimension of about 225 mm × 110 mm was developed [17]. By placing a piezoelectric cantilever with a wide fin in the wake of a cylinder blunt body, the harvester produced output power of 3 mW on a 220 kΩ resistor at wind speed of 5 m/s. Another piezoelectric energy harvester, with the maximum output power of 155 µW for a resistor of 220 kΩ when wind speed was 6.7 m/s, for autonomous wind speed sensor has been developed [18]. This harvester was successfully used to power a RF transmitter to transmit five digital words of 12-bit information every 10 s under wind speed of 6.7 m/s. The measured range of the working wind speed for this device was very narrow. Recently, MEMS piezoelectric harvesters were used to scavenge wind energy, which verified that the wind-induced vibration energy harvesters can be micromachined. A MEMS piezoelectric harvesting element, which was fixed on the free-end of a cantilevered flexible copper sheet with the dimension of 19 mm × 5 mm × 0.1 mm, produced maximum output power of 1.6 µW when wind speed was 15.9 m/s [19]. A micromachined piezoelectric wind energy harvester was directly used to scavenge wind energy, but the output power was merely 3.3 nW when wind speed was 15.6 m/s [20]. Another MEMS piezoelectric energy harvester, which realized steadily periodic wind-induced vibration when wind speed was higher than 13.2 m/s, produced maximum output power of 2.27 µW when wind speed was 16.3 m/s [1].

To protect the devices, the wind-induced vibration piezoelectric energy harvesters must be packaged when they are used to power a wireless sensor node in real applications. But the package was not considered for above piezoelectric wind energy harvesters. As the package changes the fluid field distribution around the devices, the output performance of the wind energy harvesters might be seriously affected. Therefore, the package should be considered when the harvesters were designed.

In this paper, a self-powered wireless sensor node for temperature measurement, which was powered by a wind-induced vibration piezoelectric energy harvester, was developed. Firstly, the operational mechanism of the wind energy harvester with a resonant cavity, which provides mechanical protection for the device, was analyzed. Then, the harvester was tested in a small wind tunnel to precisely evaluate its output performance. Lastly, a self-powered wireless temperature sensor node powered by the harvester was introduced and experimentally characterized.

## 2. A Wind-Induced Vibration Energy Harvester

Inspired by harmonicas, a flow-induced vibration piezoelectric energy harvester with a resonant cavity was proposed [21]. The harvester consisted of an air chamber and a piezoelectric cantilever, functioned as the reeds of harmonicas. The cantilever was mounted over a rectangular aperture on the back side of the chamber. For a prototype with the chamber diameter of 76.2 mm, cantilever length of 58 mm and cantilever width of 16 mm, the measured maximum output power on a 49.65 kΩ resistor was about 0.8 mW when air pressure in the chamber was 100 Pa. This harvester was used to convert the energy of pressured air into electrical energy. When it was directly used to scavenge ambient wind energy, the structure needs to be improved. The initial direction of the air flow is perpendicular to the top or bottom surface of the cantilever, which is obviously different from harmonicas with the initial wind speed nearly parallel to the top or bottom surface of a reed. It may cause the static wind load on the cantilever to be much larger than the dynamic wind load, and the vibration amplitude much smaller than the static deformation and relatively low electrical output. By setting the piezoelectric cantilever on the sidewall of the resonant cavity, another wind-induced vibration piezoelectric energy harvester was developed [22]. Its piezoelectric cantilever was fixed onto the front sidewall of a cuboid resonant cavity over an rectangular aperture. For a prototype with the cavity inlet of about 30 mm × 20 mm and the cantilever of 200 mm × 15 mm × 0.8 mm, the maximum output power on a 460 kΩ resistor was about 4.5 mW when the wind load produced by an electrical fan was 9.8 m/s. The size of the prototype was very large compared with a typical wireless sensor node.

**Figure 1:**
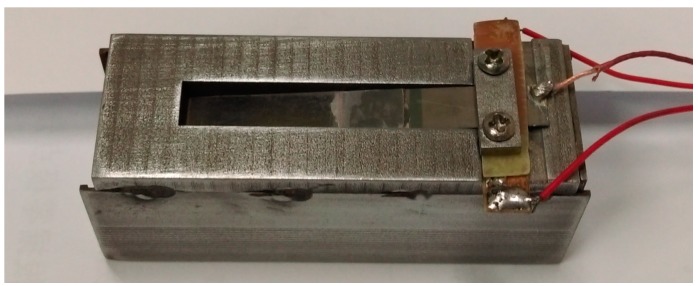
A prototype of the wind energy harvester with a resonant cavity.

After analyzing the structure of harmonicas [23,24], we designed a wind-induced vibration piezoelectric harvester, which is more similar to harmonicas, as shown in Figure 1 [11]. The wind energy harvester is mainly composed of a cantilever and a cuboid chamber. The cantilever is composed of a bimorph from Piezo Systems Corp (T215-H4-203Y, 65 Tower Office Park, Woburn, MA, USA)with the dimension of 18 mm × 6.4 mm × 0.38 mm and a flexible beam of PET plastic with the dimension of 20 mm × 6.4 mm × 0.25 mm. The front side of the chamber with the inside dimension of 62 mm × 19.6 mm × 10 mm (or the outside dimension of 64 mm × 22 mm × 14 mm) is opened as the air entrance. A rectangular aperture with the dimension of 38 mm × 6.8 mm is opened on a sidewall of the chamber as the exit. The cantilever is fixed onto the chamber at the aperture with a fixed inclined angle to form an initial attack angle. When the air flows into the chamber, wind speed decreases. According to Bernoulli’s Equation, the pressure in the chamber increases, which causes the cantilever to move upward. On the other hand, the mechanical restoring force pulls the cantilever move downward. So, when wind speed reaches a specified value, the critical wind speed, the cantilever forms a self-excited oscillation, and a periodic strain is caused in the piezoelectric layers of the bimorph. The periodic strain in the piezoelectric layer produces an electric output which can be used to power the wireless temperature sensor node. The chamber provides the mechanical protection for the cantilever and weakens the adverse influence of rain, sun light and *etc.* As a result, the working life of the harvester can be extended.

To use the above wind energy harvesters with a resonant cavity in real environments, the influence of the wind direction and wind speed on the output performance should be considered. Firstly, the harvesting efficiency is high only when the air flow blows into the chamber nearly perpendicularly to the entrance. Therefore, this harvester is especially fit for the case with constant wind direction, such as that in a ventilation duct. For the cases with changing wind direction, to obtain high power output, the harvester should be fixed on a platform which may automatically rotate to make the entrance of the harvester perpendicular to the air flow at any time. Secondly, similar to other wind-induced vibration energy harvesters, the harvester scavenges wind energy with high efficiency only when the wind speed is in a specified range. Consequently, for the case with the wind speed changing in a large range, it is better to develop an array of harvesters with different cantilevers to efficiently scavenge wind energy with different speed, just like a harmonica composed of many chambers with different reeds.

**Figure 2:**
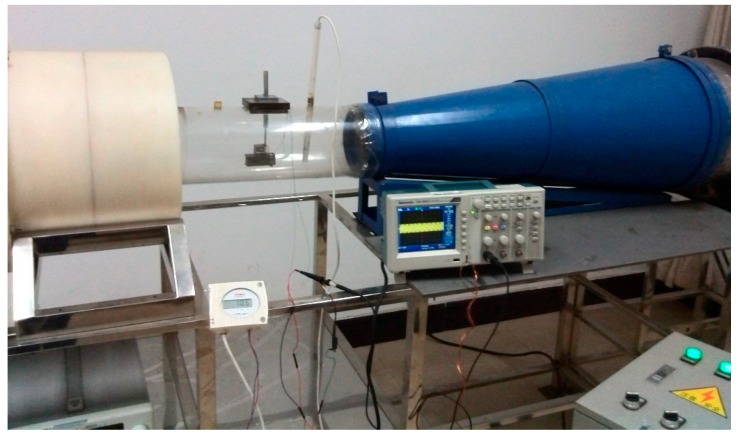
Experimental setup.

A prototype of the wind energy harvester with the initial attack angle of about 10° for the cantilever was assembled, which means that the angle between the initial wind direction and the neutral axis of the cantilever is about 10°. The properties of the prototype were measured in a small wind tunnel, as shown in Figure 2.

**Figure 3:**
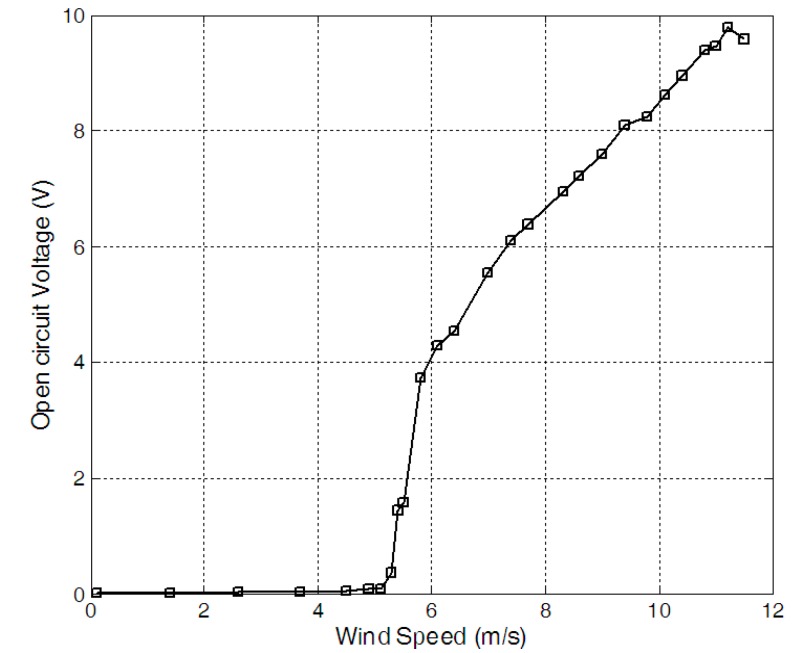
Open circuit voltage *versus* wind speed.

The measured RMS open circuit voltage *versus* wind speed was given in Figure 3. When the wind speed was lower than the critical wind speed, the electrical output of the harvester was very low. The measured critical wind speed was about 5.4 m/s. When wind speed increased from the critical wind speed, the output of the piezoelectric wind harvester increased. When the wind speed reached 11.2 m/s, the RMS open circuit voltage reached the maximum value of about 9.78 V. After that, the open circuit voltage decreased when the wind speed increased.

The load characteristics of the harvester were firstly measured when wind speed was 11.2 m/s. By connecting the harvester with a resistor of different resistances, respectively, the RMS voltages on different electrical loads were measured. And the corresponding output power on the resistors was worked outFigure 4 gave the output power. When the load resistance was 20 kΩ, the output power reached the maximum value of about 1.59 mW. After that, the RMS voltages on the resistor of 20 kΩ were recorded when the wind speed increased form 6 m/s to 11.5 m/s. The output power under different wind speed for the 20 kΩ resistor was given in Figure 5. When the wind speed was 11.2 m/s, the output power reached the maximum value of about 1.59 mW and the time history of the voltage on the resistor of 20 kΩ was given in Figure 6.

**Figure 4:**
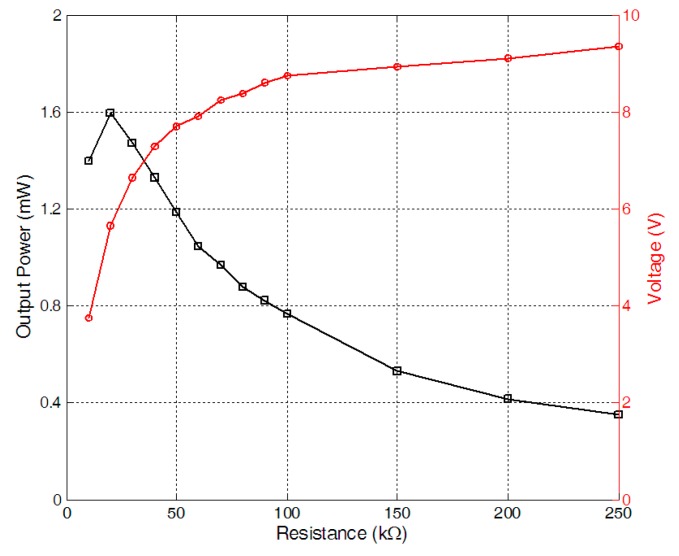
Output power *versus* resistance with wind speed of 11.2 m/s.

**Figure 5:**
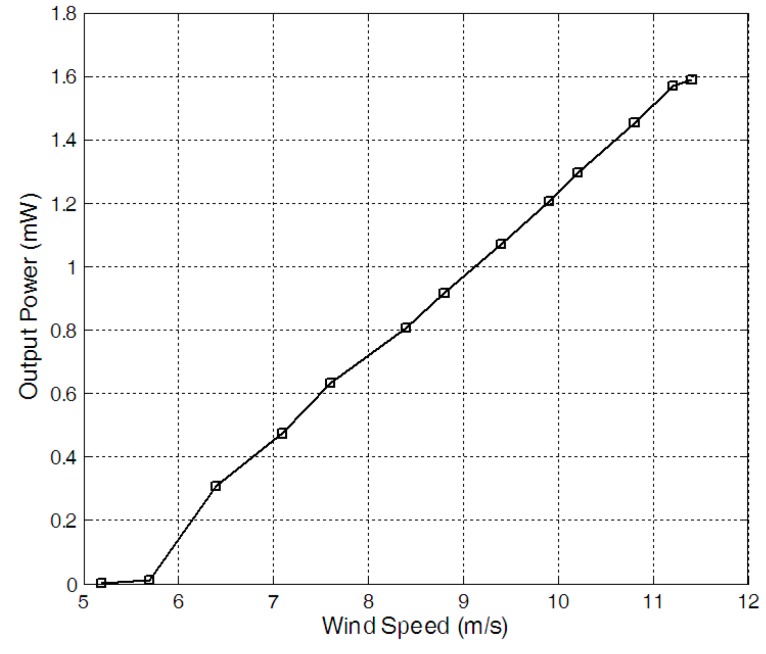
Output power on a 20 kΩ resistor *versus* wind speed.

**Figure 6:**
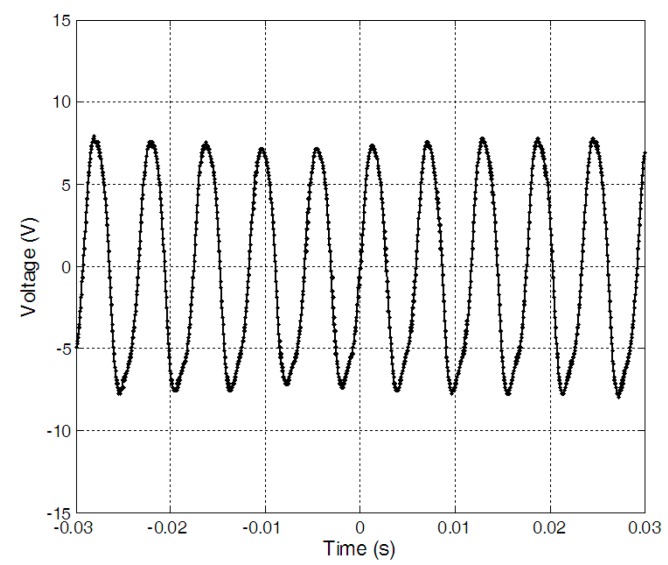
Time history of voltage on a 20 kΩ resistor with wind speed of 11.2 m/s.

The performance of the prototype was compared with several reported wind-induced vibration piezoelectric energy harvesters in Table 1. In the table, only the last four prototypes were packaged. If only the volume of the cantilever was considered in obtaining the power density, the power density of the prototype in this paper was the second high among these harvesters. The power density of the prototype developed by Weinstein *et al.* was the highest [17]. But it should be noted that there was a large fin attached onto the free end of the cantilever for this prototype and the volume of the fin was not considered in obtaining the power density [17]. In the list, only the last four prototypes were the harvesters with a resonant cavity. Among the harvesters with a cavity, the power density of the prototype in this paper was the highest whether taking the volume of the cavity into account or not.

**Table 1:** Summary of several piezoelectric wind energy harvesters.

Reference	Size	Wind Speed (m/s)	Maximum Power (µW)	Power Density (µW/cm^3^)
Tan [18]	Cantilever: 76.7 mm × 12.7 mm × 2.2 mm	6.7	155	72.3
Bryant [15]	Cantilever: 254 mm × 25.4 mm × 0.381 mm with two PZT unimorph of 46 mm × 20.6 mm × 0.254 mm Fin: 29.7 × 136 mm	7.9	2200	748 ^a^
Li [10]	Cantilever: 41 mm × 16 mm × 0.205 mm	8	260	1933
Sirohi [25]	Cantilever: 161 mm × 38 mm × 0.636 mm (2 pieces) Fin: length 251 mm, triangular section with side length of 40 mm Total: 160 mm × 250 mm	5.2	53,000	6811 ^a^
Yang [26]	Cantilever: 150 mm × 30 mm × 0.6 mm with two piezoelectric sheet of 61 mm × 35 mm × 0.5 mm Fin: 150 mm × 40 mm × 40mm	8	8400	1737 ^a^
Liu [27]	Cantilever: 3.3 mm × 2 mm × 0.4 mm (MEMS)	15.6	0.0387	14.7
He [1]	Cantilever: 10 mm × 8 mm × 0.51 mm (MEMS)	16.3	2.27	55.6
Weinstein [17]	Cantilever: 28.6 mm × 6.3 mm × 0.381 mm Fin: not given Blunt body: diameter 25 mm Total: 225 mm × 110 mm	5	3000	43,701 ^a^
Clair [21]	Cantilever: aluminum sheet of 58 mm × 16 mm × 0.3 mm with a PZT unimorph of 12 mm × 13 mm × 0.127mm Cavity: diameter of 76.2 mm, volume of 24,000 mm^3^	12.5	800	2683 ^a^ (33.3 ^b^)
Ji [22]	Cantilever: 200 mm × 15 mm × 0.8 mm with an attached piezoelectric sheet of 20 mm × 15 mm × 0.2 mm Cavity: inlet of 30 mm × 20 mm, the length is not given, which should be a little longer than 200 mm.	9.8	4500	1829 ^a^ (~37.5 ^b^)
This work	Cantilever: a PZT bimorph of 38 mm × 6.4 mm × 0.38 mm and a FET sheet of 20 mm × 6.4 mm × 0.25 mm Cavity: 62 mm × 19.6 mm × 10 mm	11.2	1590	12,780 ^a^ (130.8 ^b^)

^a^ Calculated according to the volume of the cantilever; ^b^ Calculated according to the volume of the cavity.

## 3. Self-Powered Wireless Temperature Sensor Node

**Figure 7:**
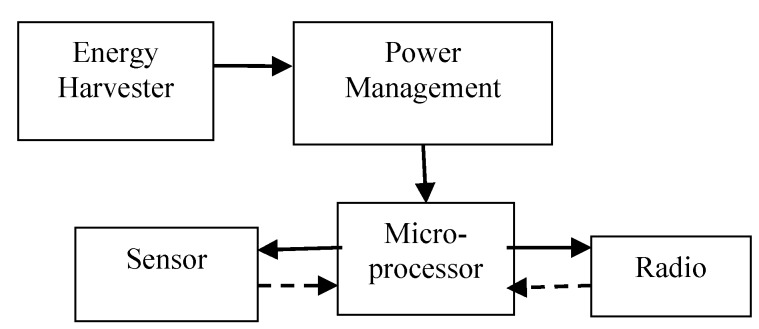
Block diagram of the self-powered wireless sensor node.

**Figure 8:**
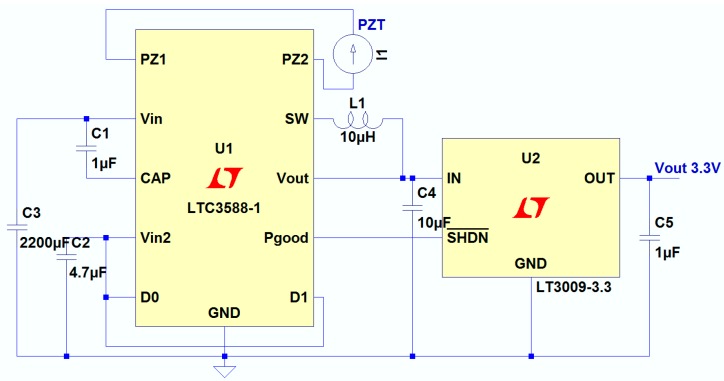
The power management circuit.

The maximum output power of the harvester was about 1.59 mW, which is too low to power a wireless sensor node directly. A power management system is needed to power the sensor, microcontroller and RF transmitter. The wireless temperature sensor node, as shown in Figure 7, consists of a wind energy harvester, MSP430 microcontroller, TMP102 temperature sensor, a power management circuit and an nRF24l01 wireless transmitting module. The piezoelectric harvester scavenges wind energy from the environment to power the node. The power management module consists of LTC3588-1 and LT3009 units, and it has three functions: rectifying the harvester’s AC output to DC voltage of 3.3 V, storing energy and switching the circuit. The output energy of the harvester will be stored in the capacitor after rectification. Once the capacitor has stored enough energy and the terminal voltage increases to the upper threshold voltage of V_high_, the system will be disconnected to the wind energy harvester to release the power from the capacitor. As the load consumes the energy, when the terminal voltage of the capacitor decreases to the lower threshold voltage of V_low_, the system disconnects it from the load and connects it to the wind energy harvester, and the charging mode begins for the next cycle. The capacitance of the capacitor was 2200 µF, and the power management circuit was shown in Figure 8. When the voltage across the energy storage capacitor is higher than the upper threshold voltage, the step-down converter is turned on and the output voltage of the LTC3588-1 is 3.6 V, and the output voltage is regulated to 3.3 V by the LT3009 linear regulator.

**Figure 9:**
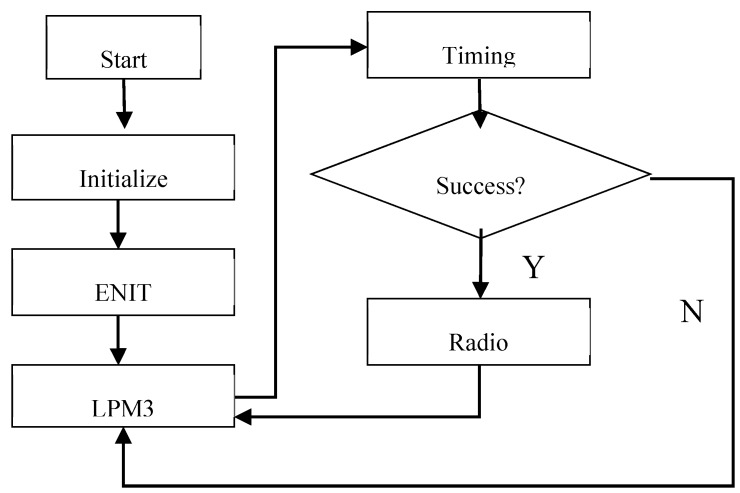
Flow chat of the measurement process.

The TMP102 temperature sensor and the MSP430 microcontroller are used to obtain the temperature, and the temperature is transmitted by the nRF24l01 transceiver. The microcontroller reads the sensor value and controls it to transmit the data to the station. To reduce the power consumption, 1 MHz of the internal oscillator DCO was chosen as the system clock and the external crystals 32,768 Hz was used as an auxiliary clock for timing. The flowchart of the measurement process was shown in Figure 9. At first, the whole system is initialized, including the system clock, Timer A0, the address, communication frequency, launch parameters and the data width of the nRF24l01 and so on. Then, the global disruptions are opened, and the wireless sensing node enters into the low power mode 3 (LPM3) [28]. After the timing is over, the temperature is measured. If the measurement successes, then the node transmits the signal and returns to the LPM3 mode after cleaning the data. If not, returns the LPM3 mode directly and waits for the next timing interrupt. In the whole test process, the microcontroller and the radio are in sleep mode at most of the time, only the timer interrupt state is in active mode, to a certain extent, reducing the system power consumption.

## 4. Experimental Results and Discussions

**Figure 10:**
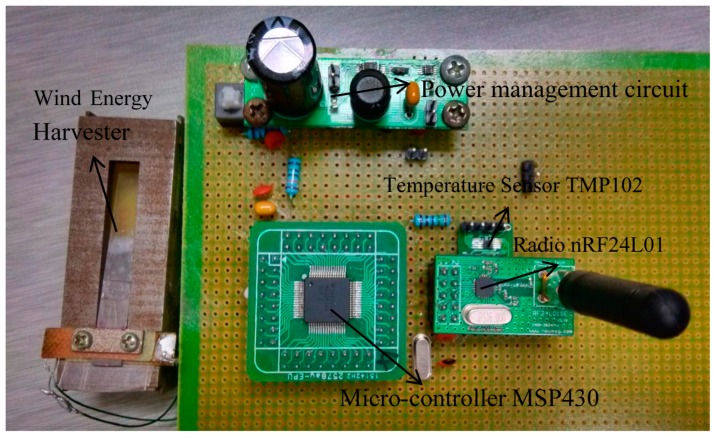
The photo of the self-powered wireless temperature sensor node.

**Figure 11:**
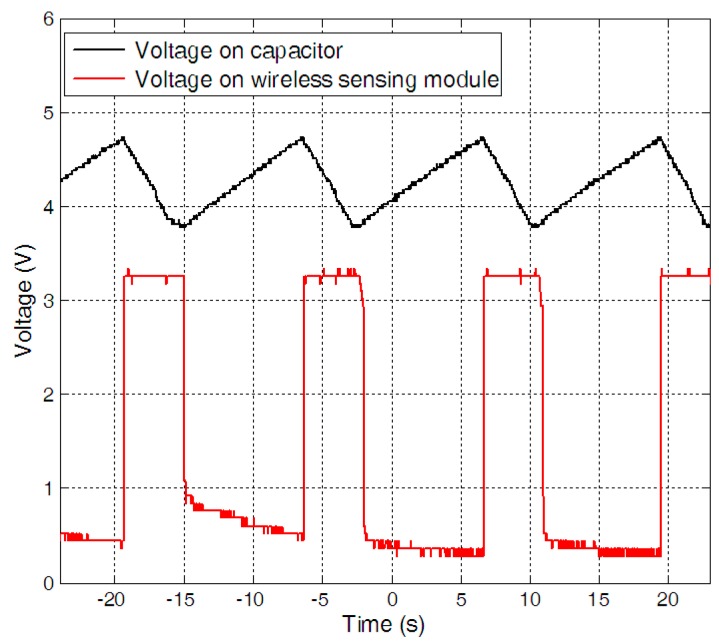
The duty time of the self-powered wireless sensor.

The performance of the self-powered wireless temperature node was tested in the small wind tunnel. Figure 10 is a photograph of the wireless temperature node powered by a wind energy harvester. When the wind speed increased from 6 m/s to 11.2 m/s, the charging time decreased. By keeping the wind speed fixed as 11.2 m/s, the performance of the wireless sensor node was evaluated. The voltages on the capacitor and the wireless sensing module were given in Figure 11. It can be found that it took about 9 s for the harvester to charge the capacitor to the upper threshold voltage of 4.78 V. As soon as the capacitor reached the upper threshold voltage, the electrical energy stored in the capacitor was discharged to the electrical load and the voltage across the capacitor decreased to the lower threshold voltage of 3.69 V in 4 s. After which, a new cycle started again.

The operational duty cycle was determined by several factors, including the size of the piezoelectric beam, the capacitance of the storage capacitor, the output voltage and the power demands of the electronics. The larger output power of the harvester, the shorter charging time; the larger storage capacitance, the longer charging time; the higher output voltage, the shorter charging time; the smaller power demands of the electronics, the lower power consume and the more powered-on time. The typical parameters of the RF transmission, measurement activity, LPM3 and temperature sensor for the wireless node were given in Table 2. The microcontroller consumes 5.28 µW and 1.386 mW during the LPM3 mode for 2000 ms, while the wireless transmission consumes 27.75 mW with a current of 7.5 mA and a voltage of 3.3 V for 10.98 ms. The system woke up and measured the temperature in about 10.74 ms, the total energy consumption for one operation was about 0.4957 mJ.

**Table 2:** Typical parameters of the wireless sensing module.

Module	Current (µA)	Voltage (V)	Power (µW)	Time (ms)
RF transmission	11,300	3.3	37,290	10.98
Measurement activity	420	3.3	1386	53.34
LPM3	1.6	3.3	5.28	2000
Temperature sensor	10	3.3	33	10.74

## 5. Conclusions

A wireless temperature node powered by a wind energy harvester was developed. The system was designed for measuring the temperature and transmits the measured data to the receiving station. The experimental results showed that the critical wind speed of the harvester was about 5.4 m/s and the output power was about 1.59 mW for the a resistor of 20 kΩ when wind speed was about 11.2 m/s. When the wind speed increased from 6 m/s to 11.5 m/s, the self-powered wireless sensor node worked normally. It took 9 s to store sufficient energy to drive the wireless temperature sensor node to work. Thus, the temperature was transmitted with the time interval of about 13s when wind speed was 11.2 m/s, and the total energy consumption for one operation was about 0.3566 mJ.
